# Decoding how higher-order network interactions shape contagion dynamics

**DOI:** 10.1007/s00285-025-02247-4

**Published:** 2025-08-19

**Authors:** István Z. Kiss, Christian Bick, Péter L. Simon

**Affiliations:** 1 Network Science Institute, Northeastern University London, London, UK; 2 Department of Mathematics, Northeastern University, Boston, USA; 3 Department of Mathematics, Vrije Universiteit Amsterdam, Amsterdam, The Netherlands; 4 Institute for Advanced Study, Technical University of Munich, Garching, Germany; 5 Department of Mathematics, University of Exeter, Exeter, UK; 6 Mathematical Institute, University of Oxford, Oxford, UK; 7 Institute of Mathematics, Eötvös Loránd University, Budapest, Hungary; 8 Alfréd Rényi Institute of Mathematics, HUN-REN, Budapest, Hungary; 9 National Laboratory for Health Security, Budapest, Hungary

**Keywords:** 34C23, 92D30

## Abstract

Complex contagion models that involve contagion along higher-order structures, such as simplicial complexes and hypergraphs, yield new classes of mean-field models. Interestingly, the differential equations arising from many such models often exhibit a similar form, resulting in qualitatively comparable global bifurcation patterns. Motivated by this observation, we investigate a generalised mean-field-type model that provides a unified framework for analysing a range of different models. In particular, we derive analytical conditions for the emergence of different bifurcation regimes exhibited by three models of increasing complexity—ranging from three- and four-body interactions to two connected populations which simultaneously includes both pairwise and three-body interactions. For the first two cases, we give a complete characterisation of all possible outcomes, along with the corresponding conditions on network and epidemic parameters. In the third case, we demonstrate that multistability is possible despite only three-body interactions. Our results reveal that single population models with three-body interactions can only exhibit simple transcritical transitions or bistability, whereas with four-body interactions multistability with two distinct endemic steady states is possible. Surprisingly, the two-population model exhibits multistability via symmetry breaking despite three-body interactions only. Our work sheds light on the relationship between equation structure and model behaviour and makes the first step towards elucidating mechanisms by which different system behaviours arise, and how network and dynamic properties facilitate or hinder outcomes.

## Introduction

**Fig. 1:**
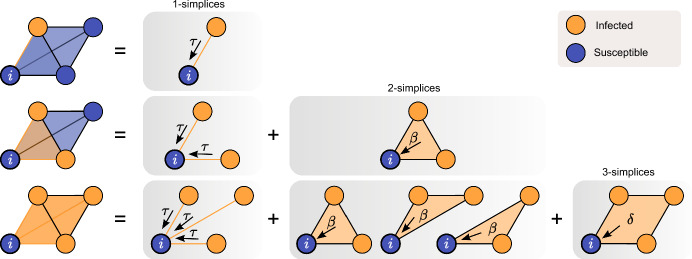
Illustration of the different possible routes of infection when higher-order interactions are modelled as simplicial complexes. The susceptible (S)  *i* is part of a 3-simplex (left column); being part of all resulting lower-order simplices. The infection pressure that *i* receives grows with the number of infectious nodes (I) within the considered simplex. Top row: One node is infectious, thus *i* can be infected by a single 1-simplex (edge) with rate $\tau > 0$. Middle row: Two nodes are infectious, thus *i* can be infected either by the two 1-simplices or by the “infectious” 2-simplex (triangle) with rate $\beta > 0$. Bottom row: All three nodes are infectious, thus *i* can be infected either by the three 1-simplices, by the three 2-simplices, or by the 3-simplex (tetrahedron) with rate $\delta >0$

The development and analysis of contagion models on higher-order networks over the last few years opened up new directions of research to understand spreading processes on networks; cf. Battiston et al. (2020), Bick et al. (2023), Ferraz de Arruda et al. (2024). In classical epidemic models on networks contagion acts along an edge $\{i,j\}$ between nodes *i* and *j*. By contrast, the probability of a susceptible node *i* becoming infected in a network with higher-order interactions depends on the order $\ell$ of a simplex/hyperedge $\{i, j_1,\dotsc j_{\ell -1}\}$ that represents a $\ell$-body interaction involving *i*. For example in case of simplicial complexes—as illustrated in Fig. 1—the rate of infection of susceptible node *i* is significantly increased by routes of infection via 2- and 3-simplices. Note that complex contagion induced by simplices/hyperedges is in addition to the pairwise contagion dynamics. While three distinct nodes *i*, *j* and *k* may be connected in a triangle of pairwise links, the existence of $\{i,j,k\}$ indicates complex contagion. If $\{i,j,k\}$ is part of a simplicial complex (i.e., it satisfies the closure relation that $\{i,j\}$, $\{j,k\}$, $\{i,k\}$ are also present), the complex contagion implies an additional infection pressure onto a susceptible node whose neighbours are infected, see Fig. 1. More generally for hypergraphs, each hyperedge exerts infection pressure that typically takes the form $\nu I^{\alpha }$ with *I* being the number of infected nodes in the hyperdege and parameters $\alpha,\nu$ (St-Onge et al. 2021, 2022; Ferraz de Arruda et al. 2020). Note that this allows for any number of infected nodes within the hyperedge to cause infection in contrast to simplicial contagion where all nodes in the simplex apart from the susceptible node have to be infectious for the excess contagion to be activated.

Complex contagion through higher-order interactions yields dynamical phenomena one may not expect if contagion is only pairwise. In contrast to traditional pairwise contagion, which is typically characterized by the disease free state losing stability to an endemic state in a forward transcritical bifurcation, contagion on higher-order networks can show hysteresis/bistability. For example, the disease-free and an endemic equilibrium may co-exist or there could be multistability between two endemic equilibria (strictly positive steady states). For example in Iacopini et al. (2019), the authors show analytically that the transition is discontinuous and that a bistable region appears where healthy and endemic states co-exist. Similarly, in Ghosh et al. (2023), the authors construct a one-dimensional model starting from an ensemble model based on the degree of the nodes. After a number of averaging assumption, their one dimensional mean-field model also displays discontinuous transition and bistability. Even more complicated behaviour is observed for contagion dynamics on hypergraphs. In Ferraz de Arruda et al. (2023), the authors show that their model has a vast parameter space, with first- and second-order transitions, bistability, and hysteresis. Finally, the authors of Kiss et al. (2023) show rigorously that the limiting mean field model of an exact complex contagion model with arbitrary simplicial complexes on a fully connected network reduces to a single differential equation which displays bistability and multistability with two strictly positive endemic steady state co-existing.

From a mathematical viewpoint, modelling of complex contagion on general structures can be done either (a) considering the entire system at once, referred to as top-down, or (b) starting at the node-level and building up to pairs, triples and so on, referred to as bottom-up (Kiss et al. 2017). For the top-down approach, the state of microscopic dynamics of susceptible/infected/susceptible (SIS) contagion model at the individual level is given by an *N*-dimensional vector of zeros and ones (Bodó et al. 2016; Kiss et al. 2023), leading to a Markov chain on a state space with $2^N$ elements. In the case of the bottom-up one starts with the evolution equations for the states of the nodes. These of course depend on the joint probabilities of pairs, the states of the pairs on the other hand depend on the states of the triples and so on. This induces a hierarchy of dependencies with the number of required equations becoming difficult to manage and analyse. In a desire to break these dependencies, higher-order moments are approximated by node-level quantities leading to so-called individual-based mean-field models (Kiss et al. 2017; Van Mieghem et al. 2008). To allow for analytical and/or numerical treatment, both the exact bottom-up and top-down models require closures. However, the bottom-up model has some advantages in that the contact structure is evident in the equations and the order at which closures are performed and the closure relationships themselves are more easily accessible. In general, closures lead to low dimensional systems of differential equations where bifurcation analysis can be employed to reveal possible behaviours and their corresponding parameter ranges.

When simplified further to allow for an explicit analysis—often to a single differential equation assuming homogeneity—many of these models show qualitatively similar behaviour at the system level. Indeed, the resulting equations are often given by polynomial vector fields whose polynomial orders relate to the interaction order and exhibit comparable global bifurcation structure. We give three concrete examples of well-known and studied models in this area and we do this by specifying explicitly the evolution of the fraction of infected individuals *y*(*t*). In Iacopini et al. (2019), the authors consider

1.1 $$\begin{aligned} {\textbf {Model A:}}\qquad \dot{y}(t)&=-\mu y(t)+\sum _{\omega =1}^L\beta _\omega \langle k_\omega \rangle y^\omega (t)[1-y(t)], \end{aligned}$$

where $\mu$ is the rate of recovery, $\beta _{\omega }$ is simplicial-order specific rate of infection, and $\langle k_\omega \rangle$ is the average number of order $\omega$ simplices a typical node is part of. Starting from the level of nodes and taking into account degrees, the authors of Ghosh et al. (2023) use a series of approximations to arrive at

1.2 $$\begin{aligned} {\textbf {Model B:}}\qquad \dot{y}(t)&=-y(t)+\lambda \beta _{\textrm{eff}}(1-y(t))y(t)+\gamma \beta _{\textrm{eff}}(1-y(t))y^{2}(t), \end{aligned}$$

which can be seen as the model above with $\omega =2$. Finally, in Kiss et al. (2023), the authors start from a fully connected network and model contagion over simplices of arbitrary order to obtain

1.3 $$\begin{aligned} {\textbf {Model C:}}\qquad \dot{y}(t)&=-\gamma y(t)+\sum _{i=1}^{M}\frac{s_{i}}{i!}\left( 1-y(t)\right) y^{i}(t), \end{aligned}$$

where $s_i$ are scaled rates of infection via *i*-simplices and $\gamma$ is the rate of recovery. From the models above, it is evident that all right hand sides are polynomials where the coefficients encode the underlying structure and the complex contagion mechanism. To put it simply we argue that even if models models A, B and C may have been derived based on different assumptions, from a mathematical viewpoint these differential equations are of the same type and can be analysed within the same framework.

Motivated by these observations, in this paper, we provide general classification results for the possible dynamical behavior of a wide class of complex contagion dynamics. Specifically, we focus on a class of vector fields that encompass many different models including Models A, B, and C above. This approach allows us to elucidate commonality between models and to map out explicitly the dependence between equation structure and model outcome. We highlight how model behaviour depends on contact network parameters including pairwise and higher-order interactions that contribute to complex contagion dynamics. We start from the simplest possible one-population model, in the sense that all nodes are topologically equivalent (meaning that on average they partake in the same number of links, 2-simplices, 3-simplices, hyperedges of various sizes and so on). This allows us to give a full mathematical characterisation of possible outcomes and outline whether critical bifurcation points depend on higher-order structure. We then move to two-population models where nodes are of two types; that is in terms of how they connect within their own population and with the other. The main findings of the paper are the identification of two distinct mechanisms of how bistability and multistability arises as well as linking system-level behaviour to constraints on the network structure.

The paper is structured as follows. In Sect. 2, we introduce the general class of equations for SIS dynamics on hypergraphs and simplicial complexes and outline how they can be derived from individual-based models. Section 3 focuses on single population models that can be reduced to a single dynamical equation and identifies what interaction order is necessary for bistability. In Sect. 4 we consider two-population models for symmetric and near-symmetric coupling. Here multistability can already arise in the presence of triplet interactions. We conclude with a discussion and identify potential future work in Sect. 5.

## Models

Consider a set of *N* nodes that we simply enumerate by $V = \{ 1,2, \dotsc, N\}$. A *hyperedge* is specified by a subset $H\subset V$; the number of distinct nodes $\ell =\vert H\vert$ is the order of a hyperedge. The set of nodes together with a set $\mathcal {H}$ of hyperedges form a *hypergraph*. Each $\ell$-uniform subhypergraph $\mathcal {H}^{(\ell )} = \{H\in \mathcal {H}: \vert H\vert =\ell \}$ that contains the edges of order $\ell$ in $\mathcal {H}$ can be identified with an adjacency tensor $h^{(\ell )}$ in the usual way: For example, $h^{(3)}_{ijk} = 1$ if $\{i,j,k\}\in \mathcal {H}$.

A *simplicial complex* is a hypergraph that satisfies the additional closure relation: If $H\in \mathcal {H}$ then for any $H'\subset H$ we have $H'\in \mathcal {H}$. In this case, the hyperedge is a *simplex*. As an example, consider for a given graph with adjacency matrix $(a_{ij})$ the simplicial complex one obtains by adding a simplex for each clique. For a three-simplex $\{i,j,k\}$ we then have $h^{(3)}_{ijk} = a_{ij}a_{jk}a_{ki}$.

### SIS dynamics of individuals

We consider SIS dynamics on hypergraphs and simplicial complexes, where the state $x_i(t)\in [0,1]$ of node *i* at time *t* corresponds to the probability of the node being infected. In classical graph-based SIS models, the infection pressure that node *j* exerts on node *i* is proportional to $a_{ij}(1-x_i)x_j$; the effect of all nodes in the neighbourhood of *i* is the sum of the individual contributions. Note that this is in fact equivalent to closing the exact individual-based model at the level of pairs, where the joint probability of a node and its neighbours is approximated by the product of node-level probabilities. For SIS dynamics on a hypergraph $\mathcal {H}$, write $\mathcal {H}_i = \{H\in \mathcal {H}\mid i\in H\}$ for the set of hyperedges that contain the node *i*. Generalizing the dynamics on graphs to include multi-body interactions, the infection pressure onto node *i* via a hyperedge $H\in \mathcal {H}_i$ is proportional to $(1-x_i) \tau _{H} \prod _{j\in H, j\ne i} x_j$, where $\tau _H$ is the infection rate corresponding to the hyperedge. Hence, the individual-based mean-field model for SIS epidemic propagation on a hypergraph takes the form

2.1 $$\begin{aligned} \dot{x}_i = - \gamma x_i+(1-x_i) \sum _{H\in \mathcal{H}_i} \tau _{H} \prod _{j\in H,j\ne i} x_j, \end{aligned}$$

for $i=1,2,\dotsc, N$, where $\gamma$ denotes the recovery rate common to all nodes.

#### Remark 2.1

First, while we consider polynomial interactions here, for a hyperedge *H* of order $\ell$, one could also consider more general interaction functions $f^{(\ell )}$. Specifically, the interaction pressure through $H\in \mathcal {H}_i$ onto *i* would be proportional to $(1-x_i) \tau _{H} f^{(\ell )}(x_H)$, where $x_H$ is the vector of states with indices in *H*.

Second, while we here restrict ourselves to fairly simple interaction pressures, note that the infection pressure can be expressed in more sophisticated ways. As an example we mention (Ferraz de Arruda et al. 2020), where the differential equation for an individual is given in the form

$$\begin{aligned} \frac{dx_i}{dt}=-\delta x_i+\lambda (1-x_i)\sum _{j:v_i\in e_j}\sum _{k=\Theta _j}^{|e_j|-1}\lambda ^*(|e_j|)\mathbb {P}_{e_j}^{v_i}(K=k), \end{aligned}$$

where $\delta$ is the rate of recovery and $\lambda$ is a background infection rate. The outer summation cycles through all the hyperedges that vertex $v_i$ belongs to. The inner summation works out the probability of the hyperedge being in state with at least $\Theta _j$ infectious nodes, this is a hyperedge specific threshold for the number of infected nodes needed to cause an infection. Finally, $\lambda ^{*}(|e_j|)$ is a cardinality-dependent rate of infection. In this formulation the infection pressure is still in the stochastic (non-mean-field) form enabling to explicitly account for the dependence on the number of infected nodes in a hyperedge.

In the following, we will focus on dynamics on hypergraphs with hyperedges up to order $\ell =4$. Thus, the hyperedges consist of (a) edges $E\subset \mathcal {H}$ of order two corresponding to two-body interactions, (b) triplets, or simply ‘triangles’, $T\subset \mathcal {H}$ of order three for the three-body interactions, and (c) quadruplets, or simply ‘squares’, $S\subset \mathcal {H}$ of order four representing four-body interactions. The sets *E*, *T*, *S* can be identified via an incidence matrix where the entry (*i*, *H*) is one if node *i* is contained in the hyperedge *H*. For example, *E* can be identified with a matrix of size $N\times|E|$ where |*E*| denotes the number of edges. If the infection rate only depends on the edge order, that is, $\tau _\ell$ is the rate for any edge of order $\ell$, the dynamical equations of *N* individual nodes in SIS dynamics on a hypergraph can be written as

2.2 $$\begin{aligned} \begin{aligned} \dot{x}_i&= \tau _2 (1-x_i) \sum _{h=1}^{\vert E\vert } E_{ih} \sum _{k\ne i} x_k E_{kh} + \tau _3 (1-x_i)\sum _{h=1}^{\vert T\vert } T_{ih} \sum _{k,l\ne i, k<l} x_kx_l T_{kh}T_{lh}\\&\quad + \, \tau _4 (1-x_i)\sum _{h=1}^{\vert S\vert } S_{ih} \sum _{k,l,m\ne i, k<l<m} x_kx_lx_m S_{kh}S_{lh}S_{mh} -\gamma x_i. \end{aligned} \end{aligned}$$

for $i=1,\ldots N$. Note that the index *h* cycles across hyperedges of a given order. In particular, in the equation above, the first summation cycles over all pairs (2-hyperedge), the second and third ones cycle over all 3- and 4-hyperedges, respectively. We give a concrete example to illustrate specific instances of these general model equations.

#### Example 2.2

A hypergraph over $N=4$ with hyperedges of order two, three and four listed below,

- $$E=\{ (1,2), (1,3), (1,4), (2,3), (2,4), (3,4)\}$$, $$|E|=6$$,
- $$T=\{ (1,2,3), (1,3,4)\}$$, $$|T|=2$$,
- $$S=\{(1,2,3,4)\}$$, $$|S|=1$$.

The first equation of the individual based model for this hypergraph takes the form

$$ \dot{x}_1 = \tau _2 (1-x_1) (x_2+x_3+x_4) + \tau _3 (1-x_1) (x_2x_3 + x_3x_4) + \tau _4 (1-x_1) x_2x_3x_4 - \gamma x_1. $$

The other equations follow in a similar way and we do not give them explicitly.

For simplicial complexes induced by an underlying graph with adjacency matrix $A=(a_{ij})_{i,j=1,\dotsc, N}$, the coefficients of the adjacency tensor can be expressed in terms of entries of $A$. Assuming that three- and four-body interactions are considered (i.e., all triangles and fully connected 4-cliques in a classical network formulation become a three- and four-body interaction, respectively), the individual based mean-field model takes the form

2.3 $$\begin{aligned} \begin{aligned} \dot{x}_i&= \tau _2 (1-x_i)\sum _{j=1}^N a_{ij}x_j + \tau _3 (1-x_i)\sum _{j=1}^N \sum _{k=j+1}^N a_{ij}a_{ik}a_{jk}x_jx_k \\&\quad + \tau _4 (1-x_i) \sum _{j=1}^N \sum _{k=j+1}^N \sum _{l=k+1}^N a_{ij}a_{ik}a_{il}a_{jk}a_{jl}a_{kl}x_jx_kx_l -\gamma x_i, \end{aligned} \end{aligned}$$

for $i=1,2,\ldots, N$. It is worth noting that in this particular example, we chose to neglect interactions among five or more nodes even if the corresponding fully connected cliques are present.

#### Example 2.3

As an example for a simplicial complex induced by a graph, consider the regular ring lattice with *N* nodes, where each node is connected to their *n* nearest neighbours. The node-level equations are given by

2.4 $$\begin{aligned} \begin{aligned} \frac{dx_i}{dt}&=\tau _2 (1-x_i)\sum _{j=1}^{n} x_{i+j}+ \tau _2 (1-x_i)\sum _{j=1}^{n} x_{i-j}\\&\quad + \, \tau _3 (1-x_i)\sum _{j=i+1}^{i+n-1}\sum _{k=j+1}^{i+n} x_jx_k + \tau _3 (1-x_i)\sum _{j=i-1}^{i-n+1}\sum _{k=j-1}^{i-n} x_jx_k\\&\quad + \, \tau _4 (1-x_i)\sum _{j=i+1}^{i+n-2}\sum _{k=j+1}^{i+n-1}\sum _{l=k+1}^{i+n} x_jx_kx_l + \tau _4 (1-x_i)\sum _{j=i-1}^{i-n+2}\sum _{k=j-1}^{i-n+1}\sum _{l=k+1}^{i-n} x_jx_kx_l-\gamma x_i, \end{aligned} \end{aligned}$$

where running indices are to be considered modulo *N*.

We note that in Example 2.3 every link is a two-hyperedge. Moreover, every three and four cliques (i.e. fully connected subgraph) is a 3- and 4-hyperdege, respectively. In this case every sub-set of a hyper-edge is also a hyper-edged. This is of course not always necessary as it is shown in Example 2.2, where despite all four nodes being in a 4-hyperedge does not imply the existence of the four possible 3-hyperedges.

### Low-dimensional reduction and examples

Since the individual-based mean-field equations for a large number of nodes *N* are challenging to analyse, further dimension reduction must be employed. This can be carried out by assuming homogeneity: Each node has the same number of neighbours and that it is part of the same number of two-body, three-body, and higher-order multi-body interactions; the actual number of interactions can be and it is usually different for different interaction orders. This allows us to reduce the *N*-dimensional system to one single differential equation and the same argument was used to derive equations including (1.1), (1.2) and, (1.3). In the following, we discuss such reductions to low-dimensional systems in more detail.

**Fig. 2:**
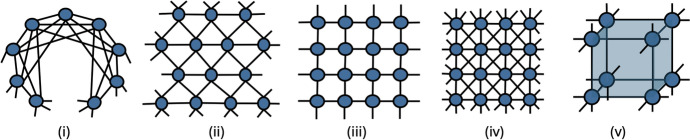
Sketches of homogenous networks with topologically equivalent nodes. From left to right we have: (i) ring lattice, (ii) triangular lattice, (iii) square lattice, (iv) square lattice with diagonals, and (iv) cube lattice with hyperedges along the faces. Periodic boundaries are assumed for each. Despite the homogeneity, the networks can be differentiated by the number of two-, three-, four-body interactions that affect each node

Homogeneity allows us to go from individual-based mean-field models to a single differential equation; we illustrate the main steps here. Let us take the ring lattice with each node connecting to *n* nearest neighbours on the right and left, see panel (i) in Fig. 2. Further we consider that each triangle and fully connected square that arises are in fact simplicial complexes of orders 2 and 3, respectively. The individual-based mean-field model for such an underlying contact network is given in (2.4). It now follows immediately that each node is contained in 2*n* one-simplices. To do the same for two-simplices, take a fixed node *i* and label nodes to the right of this with $i+1, i+2, \dots, i+n$ and to the left of it with $i-1,i-2, \dots, i-n$. We note that all these are connected to node *i*. To create a 2-simplex we need to choose two already connected nodes from the set $\{i-n, i-(n-1), \dots, i-1, i+1, i+2, \dots, i+n\}$. Choosing two nodes with two positive or two negative labels leads to $2\times n(n-1)/2$ two-simplices. Finally, choosing a node with a positive label and another one with a negative label needs to be conditioned on them being connected. For example the node labelled $i-1$, can only be connected to nodes labelled $i+1, i+2, \dots, i+(n-1)$. Following the same logic, such cross connections lead to another $n(n-1)/2$ 2-simplices, giving a total of $3n(n-1)/2$. The number of three-simplices we denote by $Q (n)$. It turns out that an explicit form for $Q (n)$ is not needed in the bifurcation analysis presented later. Moreover, if the initial conditions in all nodes are the same, all variables of the system evolve identically to one another. Thus, writing $y(t)=x_i(t)$ for each coordinate and taking into account that all nodes belong to the same number of two-body, three-body and four-body interactions, the individual level model (2.4) can be reduced to one single equation, namely

2.5 $$\begin{aligned} \frac{dy}{dt}=\tau \underbrace{(2n)}_{\text {\# of edges}}(1-y)y+\beta \underbrace{\frac{3n(n-1)}{2}}_{\text {\# of triangles}}(1-y)y^2+\delta \underbrace{Q(n)}_{\text {\# of squares}}(1-y)y^3 -\gamma y. \end{aligned}$$

This general approach can be extended to other networks as long as each node belongs to the same number of simplices of different order. By introducing *d*, *r* and *q* as the number of two-body, three-body and four-body interactions, respectively, the equation above can be genralised to

2.6 $$\begin{aligned} \dot{y}(t) = \tau d (1-y) y + \beta r (1-y)y^2 + \delta q (1-y) y^3 -\gamma y. \end{aligned}$$

This opens up the possibility to consider many different networks and it increases the range of values the rate parameters can take. Alternatively, if graphability is not an issue and stochastic simulations on an explicit network are not desired, then the system can be studied with arbitrary rates. To illustrate the flexibility of this approach, below we list further choices for $d, r$ and $q$ for a range of networks, some illustrated in Fig. 2. In no particular order options include,

(i) The ring lattice with links to the *n* nearest neighbours on left and right: $d=2n$, $r=3n(n-1)/2$, $q=Q(n)$,
(ii) Triangle lattice on a torus: $d=6$, $r=6$, $q=0$,
(iii) Square lattice on a torus: $$d=4$$, $$r=0$$, $$q=0 $$ (no four-body interactions or 3-simplices),
(iv) Square lattice with diagonals on a torus: $d=8$, $r=12$, $q=4$,
(v) Three dimensional cubic lattice with faces of the cubes as hyperedges: $d=6$, $r=0$, $q=12$.

Note that a fully connected network of size *N* has parameters $d=(N-1)$, $r={N-1\atopwithdelims ()2}$, $q={N-1 \atopwithdelims ()3}$ and corresponds to Model C. The choices above and those in models given by Eqs. (1.1), (1.2) and (1.3) are summarised in Table 1.

From a general dynamical systems point of view, the evolution equations can be seen as polynomial equations. Indeed, reordering terms, the population level dynamics can be written as

2.7 $$\begin{aligned} \frac{dy}{dt}&=(1-y)\sum _{q=1}^{Q}C_{q+1}y^{q} -\gamma y \nonumber \\&=(C_2-\gamma )y + \sum _{q=2}^{Q}(C_{q+1}-C_{q})y^{q} -C_{Q+1}y^{Q+1}, \end{aligned}$$

where the coefficients $C_q$ are typically a product between the transmission rate within a simplex/hyperedge with *q*-body interactions and the number of such simplices a typical node belongs to. However, when formulating models we find that terms are better understood if the alternative formulation in Eq. (2.6) is used. Both formulations are used throughout the paper.

**Table 1:** Interaction parameters—including the presence of higher-order interactions—determine the order and coefficients of the polynomial contagion dynamics (2.7) for homogeneous networks

	$y^1$	$y^2$	$y^3$	$y^4$
General	$C_2-{\gamma }$	$C_3-C_2$	$C_4-C_3$ or $-C_3$	$-C_4$
A	$\beta _{1}\langle k_{1}\rangle -\mu$	$\beta _{2}\langle k_{2}\rangle -\beta _{1}\langle k_{1}\rangle$	$\beta _{3}\langle k_{3}\rangle -\beta _{2}\langle k_{2}\rangle$	$-\beta _{3}\langle k_{3}\rangle$
B	$\beta _{\textrm{eff}}-1$	$\gamma \beta _{\textrm{eff}}-\lambda \beta _{\textrm{eff}}$	$-\gamma \beta _{\textrm{eff}}$	
C	$$s_1 -\gamma $$	$$ s_2/2- s_1$$	$$s_3/6-s_2/2$$ or $$-s_2/2$$	$$-s_3/6$$
(i)	$\tau \times 2n -\gamma$	$$\begin{aligned} & \beta (n(n-1)+1) \\ & \quad -\tau \times 2n \end{aligned} $$	$$\begin{aligned} & \delta \left( \frac{n(n-1)(n-2)}{3}+2\right) \\ & \quad - \beta (n(n-1)+1) \end{aligned}$$	$-\delta \left( \frac{n(n-1)(n-2)}{3}+2\right)$
(ii)	$\tau \times 6 -\gamma$	$\beta \times 6 - \tau \times 6$	$-\beta \times 6$	
(iii)	$\tau \times 6 -\gamma$	$- \tau \times 6$		
(iv)	$\tau \times 8 -\gamma$	$\beta \times 12 - \tau \times 8$	$\delta \times 4-\beta \times 12$	$-4 \times \delta$
(v)	$\tau \times 6 -\gamma$	$- \tau \times 6$	$+\delta \times 12$	$-\delta \times 12$

Rows A, B, and C correspond to models (1.1), (1.2), and (1.3), respectively. Rows (i–v) give the parameters for a (i) ring lattice, (ii) triangular lattice, (iii) square lattice, (iv) square lattice with diagonals, and (v) cube lattice shown in Fig. 2 and discussed in detail in the main text

### Multi-population models

If one assumes homogeneity in a particular population of individuals, rather than of all individuals, one obtains an equation for each population. Consider *M* populations, where $y_m\in [0,1]$ describes the probability of a typical node in population *m* to be infected and $y=(y_1, \dotsc, y_M)$ is the joint state of all populations. Write a multi-index $\textbf{n}=(n_1, \dotsc, n_M)\in \mathbb {N}^M$ of order $\vert \textbf{n}\vert = n_1+\cdots +n_M$ and set $y^\textbf{n}= y_1^{n_1}\cdots y_M^{n_M}$. Then the multipopulation SIS dynamics in a very general form are given by ordinary differential equations with polynomial vector field

2.8 $$\begin{aligned} \dot{y}_m = \sum _{\vert \textbf{n}\vert \le L}C_\textbf{n} y^\textbf{n}-\gamma y_m \end{aligned}$$

for $m=1, \dotsc, M$ and a parameter $\gamma >0$. The maximal polynomial degree $L<\infty$ that depends on the maximal order of the multibody interactions. Moreover, the coefficients $C_\textbf{n}$ are directly related to the properties of the underlying hypergraph as for $M=1$ population above.

**Fig. 3:**
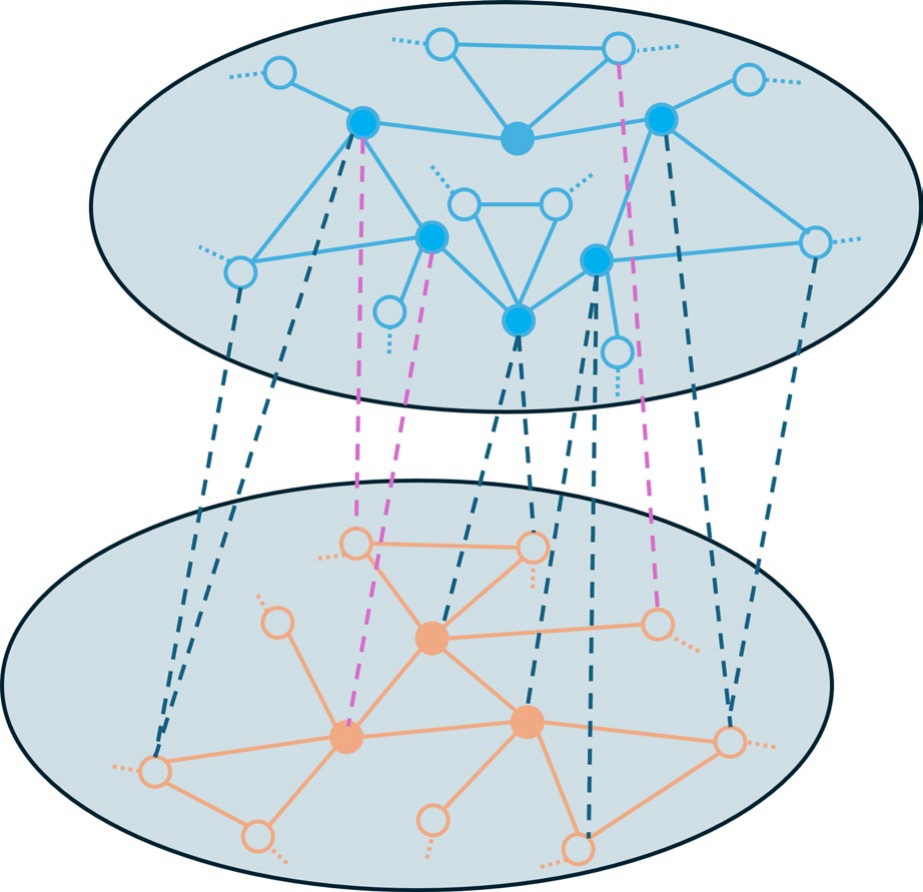
A network consisting of two populations of topologically equivalent nodes. Each node in a given population is incident to the same number of edges (two in the upper population, one in the lower) and three-body interactions (one in the top and two in the lower). Nodes with all visible neighbours are denoted by filled circles. The missing neighbours of nodes represented by unfilled circles are depicted by dotted stubs. Inter-population connections can be either pairwise (dashed cyan) or three-body (dashed blue). Three-body interactions within and between populations are depicted by thick lines. Note that, in this case, links denoting a three-body interaction do not count towards pairwise interactions. The interactions between the two populations can be modulated by considering only two-body interactions, only three-body interactions, or both types of interactions

A caricature of a network consisting of $M=2$ populations is depicted in Figure 3. We analyse bifurcations in such a two-population model explicitly in Section 4. We specifically do this for the case of two symmetrically-coupled populations, meaning that the number of links and three-body interactions are identical in both populations as well as the coupling between the two, see Section 4.1. Additionally, we will also discuss the asymmetric case in Section 4.2.

## Complete bifurcation regimes for single population models

We now focus on the simplest class of models whose dynamics are given by a single differential equation, that is, (2.8) for $M=1$. This includes networks where all nodes are topologically equivalent. We identify all possible qualitative dynamics of these models and determine the bifurcations that lead to bistability, hysteresis, multistability, and how they depend on the network parameters.

First, recall the general classification of the transcritical bifurcation and its criticality. Specifically, consider a one-dimensional equation

**Fig. 4:**
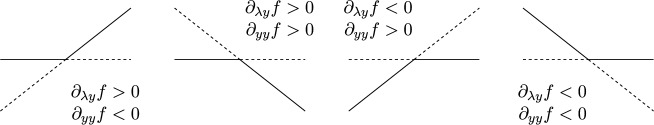
Illustration of possible transcritical bifurcation classes based on the partial derivatives of the general vector field in (3.1) at the bifurcation point. The horizontal axis represents the bifurcation parameter $\lambda$ and the vertical axis denotes the state variable *y*. Stable branches are depicted by a solid line, while unstable branches correspond to dashed lines

3.1 $$\begin{aligned} \dot{y} =f(y,\lambda ), \end{aligned}$$

that depends on a parameter $\lambda$. Assume that $y=0$ is an equilibrium for all $\lambda$, that is, $f(0,\lambda )=0$. If $\lambda ^{*}$ is a solution of $\partial _{y}f(0,\lambda )=0$ and $\partial _{\lambda y}f(0,\lambda ^{*})\ne 0$, $\partial _{y y}f(0,\lambda ^{*})\ne 0,$ then the system undergoes a transcritical bifurcation at $\lambda ^*$. Depending on the sign of the higher derivatives, the transcritical bifurcation is one of the four types depicted in Fig. 4.

### Contagion up to three-body interactions

For a one-dimensional mean-field equation, the derivatives of the vector field now determine the possible bifurcations of the disease free state. We first consider interactions given by two-body and three-body interactions.

#### **Proposition 3.1**

For a disease transmission model of the form

**Fig. 5:**
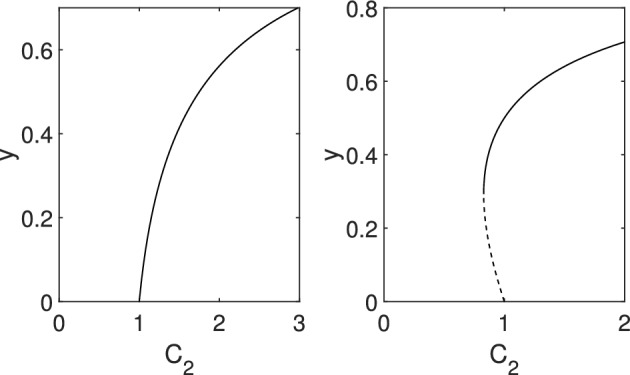
Possible outcomes for a system such as given in Eq. (3.2). Parameters are $C_3=0.5$ (left) and $C_3=2$ (right) with $\gamma =1$ in both cases. Stable and unstable branches are shown by continuous and dashed lines, respectively

3.2 $$\begin{aligned} \dot{y}=(C_2-\gamma )y+(C_3-C_2)y^2-C_3y^3 \end{aligned}$$

with $C_2\ne C_3$the disease-free steady state undergoes a transcritical bifurcation when $C_2=\gamma$. Furthermore, this can be of two types: (i) if $C_3<\gamma$ then a forward bifurcation results, and (ii) if $C_3>\gamma$ then the transcritical bifurcation is of backward type and the systems exhibits bistability as shown in Fig. 5.

#### Proof

According to the general theorem about the transcritical bifurcation and using the sign conditions in Fig. 4, we have forward transcritial bifurcation at $C_2=\gamma$, when $C_3<\gamma$. Similarly, the bifurcation is backward when $C_3>\gamma$.

The global nature of the curve can be obtained in this simple case by expressing the parameter $C_2$ in terms of $y$ as

$$ C_2= \frac{\gamma }{1-y} -C_3 y. $$

Differentiating $$C_2$$ with respect to *y* yields that the bifurcation curve has a fold point (saddle-node bifurcation) with $$C_2'(y)=0$$ when $$\gamma /C_3 = (1-y)^2$$. This fold point lies in the relevant domain ($$0<y<1)$$ when $$C_3>\gamma $$, i.e., when the transcritical bifurcation is of backward type. This completes the proof of the statement. $$\square $$

#### Remark 3.2

If $C_2=C_3$, then the quadratic term vanishes, leading to a $y\mapsto -y$ symmetry. This means that the trivial state loses stability in a pitchfork bifurcation. Furthermore, this is an example of how three-body interactions can change the type of pitchfork bifurcation; cf. Kuehn and Bick (2021).

Proposition 3.1 now allows us to classify the dynamics in terms of the network parameters. For a ring lattice as given in Eq. (2.5) with infection through triangles or 2-simplices, we consider $\tau$ as the bifurcation parameter. Rearranging the equation as per Proposition 3.1 above leads to

3.3 $$\begin{aligned} \dot{y} =(\tau \times 2n-\gamma )y + \left( \beta \times \frac{3n(n-1)}{2}-\tau \times 2n \right) y^2-\beta \times \frac{3n(n-1)}{2}y^3 \end{aligned}$$

According to Proposition 3.1, the bifurcation occurs when $2\tau n=\gamma$ with bistability if

3.4 $$\begin{aligned} 3\beta n(n-1)-2\gamma >0, \end{aligned}$$

which is equivalent to

3.5 $$\begin{aligned} n(n-1)>\frac{2}{3}\frac{\gamma }{\beta }. \end{aligned}$$

For fixed transmission rates through links and 2-simplices as well as fixed recovery, the condition above gives a constraint on the density of links, and implicitly 2-simplices, needed for bistability. The immediate interpretation of this result is that the same disease unfolding on communities with different contact patterns can lead to different outcomes.

Our result can also be used to re-derive the bifurcation conditions for the models cited in Sect. 2.2. For example, for the model up to 2-simplices in Kiss et al. (2023), our Proposition predicts that the bifurcation occurs at $C_2=\gamma$, that is $\lambda =\gamma$. For bistability our Proposition requires $C_3>\gamma$ which translates to $\mu /2<\gamma$ which is exactly as reported in Kiss et al. (2023).

### Contagion up to four-body interactions

We now consider interactions given by two-body, three-body and four-body interactions and determine the bifurcations. The different possibilities for the transcritical bifurcation characterize the possible contagion dynamics.

#### **Proposition 3.3**

For a disease transmission model of the form

**Fig. 6:**
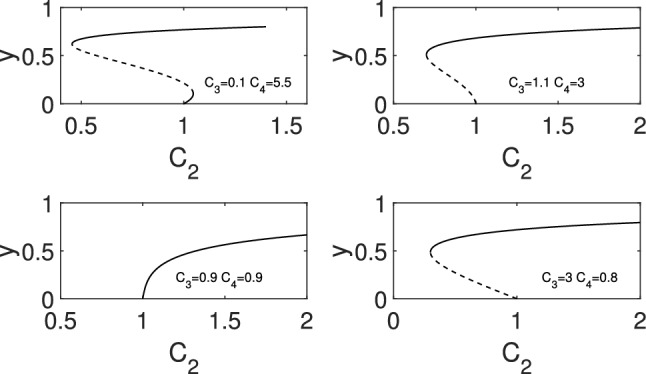
Possible bifurcation behaviour of the disease free steady state and emergent nontrivial equilibrium branches for (3.6). Parameter values are given in each panel and $\gamma =1$ for all cases. Stable and unstable branches are shown by continuous and dashed lines, respectively

3.6 $$\begin{aligned} \dot{y} =(C_2-\gamma )y+(C_3-C_2)y^2+(C_4-C_3)y^3-C_4y^4 \end{aligned}$$

with $C_2\ne C_3$ the disease-free steady state undergoes a transcritical bifurcation when $C_2=\gamma$. This can be of two types: (i) if $C_3<\gamma$ then a forward bifurcation results, and (ii) if $C_3>\gamma$ then the transcritical bifurcation is of backward type. The systems exhibits three different global bifurcation curves:

- *If *$C_3>\gamma$, then the bifurcation curve has a fold point leading to bistability between the disease-free and the endemic steady state for certain values of the parameter $C_2$, see top- and bottom-right panels in Fig. 6.
- *If *$C_3<\gamma$ and $C_4<\gamma$, then the bifurcation curve has no fold point, hence either the disease-free or the endemic steady state is stable for any value of the parameter $C_2$, see bottom-left panel in Fig. 6.
- *If *$C_3<\gamma$ and $C_4>\gamma$, then the bifurcation curve has two fold points, leading to two stable endemic branches. That is, bistability may occur between two endemic steady states for certain values of the parameter $C_2$, see top-left panel in Fig. 6.

The different cases are shown in Fig. 6. We note that two subfigures are shown for the case $C_3>\gamma$. The difference between them is in the value of $C_4$, namely, there is an inflection point on the unstable branch when $C_4>\gamma$.

#### Proof

According to the general theorem about the transcritical bifurcation and using the sign conditions in Fig. 4, we have forward transcritial bifurcation at $C_2=\gamma$, when $C_3<\gamma$. Similarly, the bifurcation is backward when $C_3>\gamma$.

The global nature of the curve can be obtained in this simple case by expressing the parameter $C_2$ in terms of $y$ as

$$ C_2= \frac{\gamma }{1-y} -C_3 y - C_4 y^2. $$

Differentiating $C_2$ with respect to *y* yields that the bifurcation curve has a fold point, $C_2'(y)=0$, when $C_3 +2C_4 y = \gamma / (1-y)^2$. The shape of the bifurcation curve depends on the second derivative $C_2''(y)$ as well. It has an inflection point when $\gamma /C_4 = (1-y)^3$. That is we have an inflection point when $C_4>\gamma$, leading to the bifurcation curves shown in Fig. 4 and completing the proof of the statement. $\square$

We note that an important feature of such models is that the transcritical bifurcation point is not affected by the inclusion of the higher-order contagion terms. For both the cubic and quartic case, the transcritical bifurcation occurs at $C_2=\gamma$. However, the inclusion of the higher-order infection term changes the global bifurcation picture and leads to bistability, a phenomenon that is not observed in classical susceptible-infected-susceptible models.

We now use this result to determine the bifurcation behaviour for different networks. Specifically, consider the one-dimensional Eq. (2.6) which corresponds to Eq. (3.6) with parameters $C_2=\tau d$, $C_3 = \beta r$, and $C_4= \delta q$. Fix the epidemic parameters such that

$$ \frac{1}{12}< \beta< \frac{1}{6}, \qquad \frac{1}{12}< \delta < \frac{1}{4}\,\,\, \text {and} \,\,\, \gamma =1. $$

For the square lattice with diagonals on a torus we have $d=8$, $r=12$, $q=4$. Thus, $C_3=12 \beta >1$ and $C_4=4 \delta <1$ and the bifurcation diagram is like that in the bottom right of Fig. 6. That is bistability occurs between the disease-free and the endemic steady state.

The triangle lattice on a torus is characterized by $d=6$, $r=6$, $q=0$. This implies $C_3=6 \beta <1$ and $C_4=0 <1$, and thus the bifurcation diagram is like that in the bottom left of Fig. 6. That is, there is no bistability for this network.

For the three dimensional cubic lattice with squares as hyperedges we have $d=6$, $r=0$, $q=12$. Thus, $C_3=0 <1$ and $C_4=12 \delta >1$. The bifurcation diagram is like that in the top left of Fig. 6. That is bistability may occur between two endemic steady states.

We note that introducing higher order contagion up to $Q$-body interactions, as shown in Eq. (2.7), the degree of the polynomial in *y* will be $Q+1$. The coefficients of such a polynomial can have alternating signs if the infection rates for different hyperedge sizes are chosen appropriately. Then applying the Cartesian rule for the existence of positive roots of the polynomial, there can be $Q$ positive steady states, corresponding to the positive roots of the polynomial. That is, the higher the order of contagion, the more complicated the bifurcation structure of the dynamics can be.

## Bifurcation regimes for two-population networks

Rather than considering a homogeneous population of nodes, we now shift the focus to networks that consist of two distinct populations, each consisting of topologically equivalent nodes. More precisely, consider a network with two populations of nodes—illustrated in Fig. 3. Within population 1, each node has $d_1$ edges and $h_1$ triangles as well as $d_2$ edges to nodes in population 2. Within population 2, each node has $d_4$ edges and $h_2$ triangles as well as $d_3$ edges to nodes in population 1. For homogeneous initial conditions, this results in the evolution of the probability of infection $y_j$ of a node in population *j* given by

4.1a $$\begin{aligned} \dot{y}_1&= \tau (1-y_1) (d_1y_1 + d_2 y_2) +\beta h_1 (1-y_1) y_1^2-\gamma y_1, \end{aligned}$$

4.1b $$\begin{aligned} \dot{y}_2&= \tau (1-y_2) (d_3y_1 + d_4 y_2) +\beta h_2 (1-y_2) y_2^2 -\gamma y_2. \end{aligned}$$

This is a specific case of the general model Eq. (2.8) for $M=2$ populations.

In this section, we consider the possible bifurcation diagrams in the $(\tau, I)$-plane, where $I=N_1 y_1 + N_2 y_2$ with $N_i$ denoting the number of nodes in population *i*. In contrast to one-population networks, where multistability requires the existence of 3-simplices (cf. Sect. 3), we show that multistability is already possible with 2-simplices (triangles)—at the expense of a more complicated network structure. We first consider two symmetrically coupled populations and then analyse the dynamics if the symmetry is broken.

### Two symmetrically coupled populations

Assume symmetric coupling, that is, $d_1 = d_4$, $d_2=d_3$, and $h=h_1=h_2$. Then the system has a $\mathbb {Z}_2$-symmetry that acts by permuting the indices of the populations. The symmetry implies that the set where the states of both populations are equal, $\Delta:= \{y = y_1 = y_2\}$, is dynamically invariant; cf. Golubitsky and Stewart (2002). Thus, to analyse the dynamics of (4.1), we can first consider the dynamics on $\Delta$ and then the dynamics transverse to $\Delta$.

#### Dynamics and bifurcations in symmetric systems

To simplify notation, we will first consider the system

4.2a $$\begin{aligned} \dot{y}_1&= C_1y_1 + C_1'y_2 + C_2y_1^2 + C_2'y_1y_2 + C_3 y_1^3 \end{aligned}$$

4.2b $$\begin{aligned} \dot{y}_2&= C_1y_2 + C_1'y_1 + C_2y_2^2 + C_2'y_2y_1 + C_3 y_2^3. \end{aligned}$$

with a polynomial vector field. Note that the SIS model (4.1) is a special case with $C_1 = \tau d_1-\gamma$, $C_1' = \tau d_2$, $C_2 = -\tau d_1+\beta h_1$, $C_2' = -\tau d_2$, and $C_3 = -\beta h_1$. Note that any point in $\Delta$ is a *symmetric state*, that is, the state of both populations take the same value. Any point in $\mathbb {R}^2\smallsetminus \Delta$ we refer to as an *asymmetric state*.

First, we will consider the dynamics of symmetric states within $\Delta$; these behave like a single population (cf. Sect. 3.1). Specifically, the dynamics on $\Delta$ evaluate to

4.3 $$\begin{aligned} \dot{y}&= (C_1+C_1')y + (C_2+C_2')y^2 + C_3 y^3. \end{aligned}$$

If $C_2+C_2'\ne 0$, the trivial equilibrium $y = 0$ undergoes a transcritical bifurcation at $C_1+C_1' = 0$ within $\Delta$. The emergent branch of nontrivial equilibria is determined by the quadratic equation

4.4 $$\begin{aligned} C_1+C_1' + (C_2+C_2')y + C_3 y^2 = 0. \end{aligned}$$

Depending on the parameter values there may or may not be a fold point for $y>0$.

Second, we can determine dynamics and bifurcations close to these equilibria transverse to $\Delta$ to get insights into the full dynamics of (4.2). Linear stability at any point $(y_1, y_2)= (y,y)\in \Delta$ is given by

4.5 $$\begin{aligned} A^{\Delta } = \left( \begin{array}{cc} C_1+(2C_2+C_2')y+3C_3y^2& \quad C_1'+C_2'y\\ C_1'+C_2'y & \quad C_1+(2C_2+C_2')y+3C_3y^2 \end{array}\right). \end{aligned}$$

Naturally, the symmetry constrains the eigenvalues and eigenvectors of $A^{\Delta }$ given by

4.6a $$\begin{aligned} \lambda ^\parallel (y)&= C_1+(2C_2+C_2')y+3C_3y^2+C_1'+C_2'y,&v^\parallel&= (1,1)^T \end{aligned}$$

4.6b $$\begin{aligned} \:\:\:\:\:\:\lambda ^\perp (y)&= C_1+(2C_2+C_2')y+3C_3y^2-C_1'-C_2'y,&v^\perp&= (1,-1)^T. \end{aligned}$$

The first eigenvalue corresponds to linear stability with respect to perturbations within $\Delta$ that reduce to the one-dimensional system discussed above. The second eigenvalue on the other hand corresponds to linear stability with respect to perturbations transverse to $\Delta$. Its zeros indicate bifurcations that give rise to nontrivial equilibria off $\Delta$. Due to the symmetry, such bifurcations are generically of pitchfork type.

We can compare the two eigenvalues to get insight into the relationship of bifurcations within and transverse to $$\Delta $$. Note that for $$0\le y \le 1$$, the sign of $$C_1'+C_2'y$$ determines whether $$\lambda ^\parallel \le \lambda ^\perp $$ or $$\lambda ^\parallel \ge \lambda ^\perp $$. Specifically, if $$C_1'+C_2'y=\tau d_2 (1-y)\ge 0$$, we have $$\lambda ^\perp \le \lambda ^\parallel $$ and the stability of any equilibrium within $$\Delta \:$$$$(\lambda ^\parallel <0)$$ implies stability in the full system (4.2). This also means that any stable equilibrium within $$\Delta $$ first loses stability within $$\Delta $$ (in a transcritical bifurcation) before it can lose stability in the transverse direction.

#### Equilibria and bifurcations of symmetric states

The first step to gain insights into the SIS dynamics (4.1) is to analyze the dynamics and bifurcations within the invariant subspace $\Delta$. The trivial equilibrium undergoes a transcritical bifurcation at $0= \tau ( d_1+d_2)-\gamma = C_1+C_1' = 0$ or

4.7 $$\begin{aligned} \tau = \tau _{tr}= \frac{\gamma }{d_1+d_2} \end{aligned}$$

if $C_2+C_2' = \beta h_1-\gamma \ne 0$. Moreover, the sign of $\beta h_1-\gamma$ (the quadratic term in (4.3)) determines the type of transcritical bifurcation: It is of forward type when $\gamma > \beta h_1$ and it is of backward type for $\gamma < \beta h_1$.

The nontrivial branch of equilibria (4.4) is given by

4.8 $$\begin{aligned} \tau = \frac{\gamma }{(d_1+d_2)(1-y)} - \frac{\beta h_1}{d_1+d_2}y. \end{aligned}$$

We can see that $\tau \rightarrow \infty$ as $y\rightarrow 1$. As the solution to a quadratic equation, the type of bifurcation of the trivial equilibrium also determines bistability: There is a fold point for $0<y<1$ if $\gamma < \beta h_1$. In this case, bistability occurs, i.e., for certain values of $\tau$ the larger endemic steady state is stable together with the trivial steady state $y=0$. The following theorem summarizes the results about the bifurcation diagram of the symmetric model.

##### **Theorem 4.1**

Transcritical bifurcation occurs in two symmetric populations (4.2) at $\tau = \tau _{tr}$ given in (4.7). The shape of the bifurcation curve of non-trivial steady states depends on the parameters that determine the higher-order interactions as follows.

- If $\beta h_1 < \gamma$, then the$(\tau, y)$ bifurcation diagram is of forward type and the non-trivial steady state is stable.
- If $\gamma < \beta h_1$, then the $(\tau, y)$bifurcation diagram is of backward type and it has a fold point. The non-trivial steady state on the lower branch (below the fold point) is unstable, while the non-trivial steady state on the upper branch (above the fold point) is stable.

**Fig. 7:**
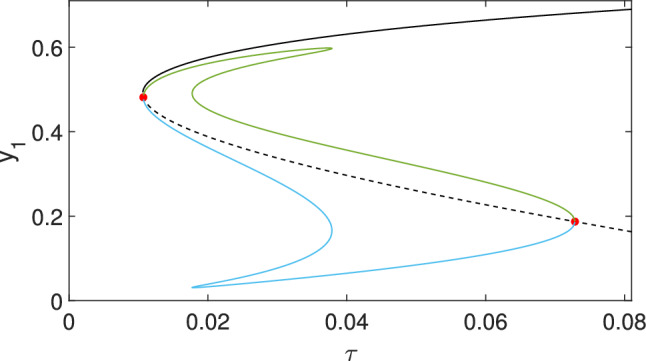
Branches of equilibria in the ($\tau, y_1$)-plane for two symmetric populations (4.2) with parameters $d_1=4$, $d_2=3$, $h=1$, $\beta =3.85$, and $\gamma =1$. The black line corresponds to the symmetric equilibrium branch in $\Delta$; a solid line corresponds to a stable equilibrium, a dashed line to an unstable solution. Change of transverse stability occurs at pitchfork bifurcations indicated by red dots. The emergent asymmetric steady states are depicted by green and blue curves (stability not shown)

**Fig. 8:**
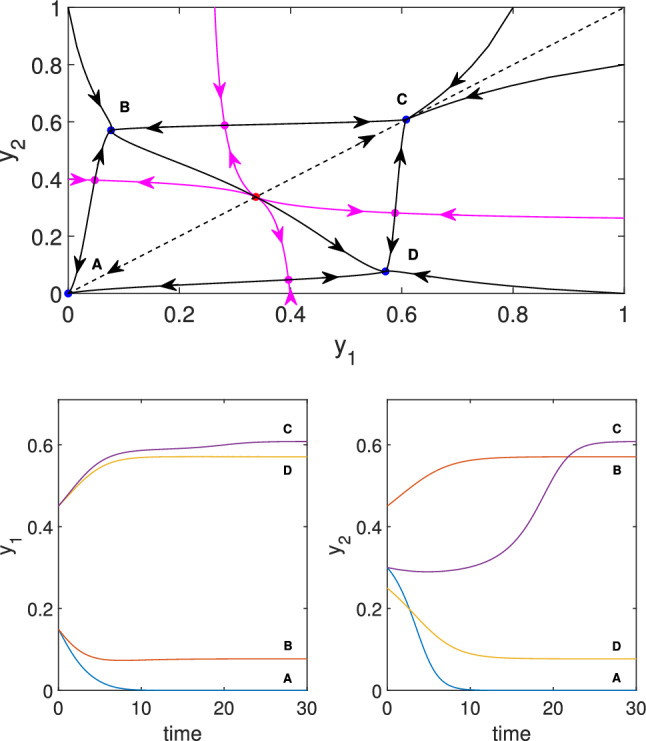
Illustration of the behaviour of the symmetric system (4.2) with parameters $d_1=4$, $d_2=3$, $\gamma =1$, $\beta =3.85$, $h=1$, and $\tau =0.03$. Top: Phase plane with stable nodes A, B, C, D (blue dots), unstable symmetric equilibrium (red dot), and four additional (unstable) saddle equilibria (magenta dots). Bottom: Time evolution of $y_1$ and $y_2$ of solutions converging to one of the four labelled stable equilibria

The statement of the Theorem can be translated to the language of phase portraits as follows. The number and stability of symmetric steady states (i.e., $y=y_1=y_2$) is determined by the theorem. In the first case, when $\beta h_1 < \gamma$, there are two ranges of the bifurcation parameter $\tau$. If $\tau < \tau _{tr}$, then the only symmetric steady state is the disease-free one, $y_1=y_2=0$, and it is a globally stable node. If $\tau > \tau _{tr}$, then the disease-free steady state is unstable and there is a globally stable non-trivial symmetric steady state $y_1=y_2\ne 0$. In the second case, when $\beta h_1 > \gamma$, there are three ranges of the bifurcation parameter $\tau$, see Fig. 7. Denoting by $\tau _f$ the fold (or turning) point of the bifurcation curve, the first region is $\tau < \tau _{f}$, where the only symmetric steady state is the disease-free one $y_1=y_2=0$, and it is a globally stable node. In the second region, $\tau _{f}< \tau < \tau _{tr}$, there are three symmetric steady states: the disease-free $y_1=y_2=0$, an unstable non-trivial steady state, which is a saddle and a stable non-trivial steady state, which is a stable node. Finally, in the third region, $\tau > \tau _{tr}$, the disease-free steady state is unstable and there is a globally stable non-trivial symmetric steady state $y_1=y_2\ne 0$. Thus the bifurcation diagram in Fig. 7 translates to three different phase portraits. These are not shown here since the non-symmetric steady states, which also appear in the phase portrait, will be determined below. We refer to Fig. 8 where the phase portraits for the case of $\tau _{f}< \tau < \tau _{tr}$ is presented together with the non-symmetric steady states.

#### Pitchfork bifurcation towards non-symmetric steady states

In a second step, we now analyse potential bifurcation points of the nontrivial branch of symmetric equilibria (4.8) within $\Delta$ in the transverse direction. For the SIS dynamics, the bifurcation condition for the general expression of the transverse eigenvalue (4.6b) evaluates to

4.9 $$\begin{aligned} \tau (d_1-d_2)-\gamma +2(\beta h_1-\tau d_1)y-3\beta h_1 y^2 = 0. \end{aligned}$$

Eliminating $\tau$ from (4.8) to (4.9) gives that the *y*-value of the pitchfork point has to satisfy

4.10 $$\begin{aligned} f(y):= 2\gamma d_2 + (d_1-d_2)\gamma y -(d_1+3d_2)\beta h_1y (1-y)^2 = 0. \end{aligned}$$

The goal now is to find a solution $y\in [0,1]$ for which (4.8) yields a positive value of $\tau$, i.e., $\gamma - \beta h_1 y (1-y) >0$.

Depending on the values of the parameters, the function *f* may have zero or two roots in the interval [0, 1]. At the endpoints of the interval, we have $f(0)=2\gamma d_2 >0$ and $f(1)=\gamma (d_1 +d_2)>0$. Then the bifurcation conditions for the appearance of two roots is $f(y)=0$ and $f'(y)=0$, that is,

4.11 $$\begin{aligned} 2\gamma d_2 + (d_1-d_2)\gamma y -(d_1+3d_2)\beta h_1y (1-y)^2&= 0,\end{aligned}$$

4.12 $$\begin{aligned}\:\:\:\:\: (d_1-d_2)\gamma -(d_1+3d_2)\beta h_1(1-3y)(1-y)&= 0. \end{aligned}$$

Dividing both equations by $d_2$ we can observe that the single parameter $d=d_1/d_2$ determines the existence of the pitchfork bifurcation point instead of the two parameters $d_1$ and $d_2$. Dividing the first Eq. (4.11) by the second (4.12), we can express the new parameter $d$ in terms of $y$ as

$$ \frac{d_1}{d_2} = d=\frac{1-3y+y^2}{y^2}. $$

Then substituting this expression into the second Eq. (4.12), one can determine the other parameters as

$$ \beta h_1= \frac{\gamma }{(1-3y+4y^2)(1-y)}. $$

It is easy to check that the bifurcation curve, parameterised by the above two equations (note that $y$ is the independent variable) is monotone in the $(d,h_1)$ plane. Pitchfork bifurcation points, and hence symmetry breaking bifurcation, occur along this curve. The implicit relation between $d$ and $h_1$ given by the parametrisation in terms of $y$ can also be expressed as a direct relation between the two variables, namely we can introduce a function $S$ such that $S(d)=h_1$. Using this curve, we can complete the characterisation of the bifurcation diagrams in the $(\tau, y)$-plane as it is presented in the theorem below.

##### **Theorem 4.2**

*The parameter plane *$(d_1/d_2, h_1)$* can be divided according to the shape of the *$(\tau, y)$* bifurcation diagram as follows*.

- *If *$h_1 < \gamma /\beta$, * then the *$(\tau, y)$* bifurcation diagram is of forward type and there is no pitchfork point*.
- *If *$\gamma /\beta< h_1 < S(d_1/d_2)$, *then the *$(\tau, y)$* bifurcation diagram is of backward type and there is no pitchfork point*.
- *If *$$ S(d_1/d_2) < h_1$$, * then the *$$(\tau, y)$$* bifurcation diagram is of backward type and there are two pitchfork points, and two branches of non-symmetric solutions*.

#### Nonsymmetric solutions of the symmetric equation

Can there be bistability between two nonsymmetric endemic steady states for the SIS dynamics (4.1)? The pitchfork bifurcations discussed above give rise to nonsymmetric steady states, but these may not necessarily be stable close to the bifurcation point.

Indeed, the first result shows that equilibria emerging in the pitchfork bifurcations are unstable. Recall that for equilibria $y\in \Delta$ with $0\le y < 1$ the eigenvalues of the linearization satisfy $\lambda ^\perp \le \lambda ^\parallel$. This means that the transverse bifurcation condition $\lambda ^\perp = 0$ can only be satisfied if $\lambda ^\parallel > 0$. In summary we have

##### **Proposition 4.3**

*For the SIS dynamics* (4.1), *any pitchfork bifurcation of the symmetric branch of equilibria* (4.8) *within* $\Delta$*is subcritical. That is, the emerging branch of nonsymmetric equilibria are unstable near the bifurcation point*.

Thus, secondary bifurcations along the branch are necessary for bistability between nonsymmetric equilibria to emerge. To get insight where these bifurcations may appear, we compute the branch of asymmetric equilibria in the $(\tau, y_1)$-plane. Specifically, equilibria of (4.1) for symmetric coupling satisfy

4.13a $$\begin{aligned} \tau (d_1y_1 + d_2 y_2)&= \frac{\gamma y_1}{1-y_1} - \beta h_1 y_1^2, \end{aligned}$$

4.13b $$\begin{aligned} \tau (d_2y_1 + d_1 y_2)&= \frac{\gamma y_2}{1-y_2} - \beta h_1 y_2^2. \end{aligned}$$

Dividing the first equation by the second one, we can eliminate $\tau$ and obtain a relation between $y_1$ and $y_2$ as

$$ (d_1y_1 + d_2 y_2) \left( \frac{\gamma y_2}{1-y_2} - \beta h_1 y_2^2 \right) = (d_2y_1 + d_1 y_2) \left( \frac{\gamma y_1}{1-y_1} - \beta h_1 y_1^2 \right). $$

This can be rearranged to

$$ \gamma \frac{d_2(y_1+y_2) + (d_1-d_2)y_1y_2}{(1-y_1)(1-y_2)} = \beta h_1 \left( (d_1-d_2)y_1y_2 + d_2 (y_1+y_2)^2 \right). $$

Dividing this equation by $\gamma d_2$ and writing $d=d_1/d_2$, $B= \beta h_1/\gamma$, $x=(y_1+y_2)/2$, and $z=y_1y_2$, yields

$$ \frac{2x + (d-1)z}{1-2x+z} = B ((d-1)z +4x^2). $$

This now enables us to express $y_2$ in terms of $y_1$ and compute the $(\tau, y_1)$ branch parametrised by $x$. Specifically, choosing a value of $x$ (the proper interval where $x$ is varied will be defined later), the value of $z$ can be determined from the above equation which is a quadratic one in $z$. Once $x$ and $z$ are given, the values of $y_1$ and $y_2$ are determined from the system below

$$ y_1+y_2 = 2x, \qquad y_1y_2 = z, $$

as

$$ y_{1,2} = x \pm \sqrt{x^2-z}. $$

It is easy to check that both roots are real and are in the interval [0, 1] if and only if

$$ 2x-1< z< x^2 <1 \quad \text{ and } \quad x>0 \quad \text{ and } \quad z>0 $$

hold. Hence $x$ is varied in the interval [$1/2,1$] and this is restricted further by the upper and lower bound conditions on *z*.

As these equations are typically not accessible analytically, we can compute the $(\tau, y_1)$ solution curve numerically for fixed parameters $d_1$, $d_2$, $h$, $\beta$ and $\gamma$. Here we show a concrete example rather than attempting a full characterization. Fig. 7 shows a situation corresponding to the third case in Theorem 4.2, when there are two pitchfork points. The non-symmetric steady states are computed by using the algorithm detailed above. Numerical computation shows that the branch of non-symmetric steady states connects the two pitchfork points, as it is shown in Fig. 7. Moreover, it turns out that this branch might have an *S*-shape, hence there are five regions of $\tau$ that yield different phase portraits. In the third region for $\tau$ we can have six non-symmetric steady states, hence the total number of steady states is nine. Two symmetric steady states (blue dots in Fig. 8) are stable nodes, and one is an unstable node (red dot). Four of the non-symmetric ones are saddles (magenta dots) and two of them are stable nodes (blue dots B and D). The phase portrait is shown in the top panel of Fig. 8. As we can see, there are four stable steady states (blue dots), and the boundary between their basins of attraction are formed by the stable manifolds of the saddle points (shown with magenta dots). The time dependence is shown in the bottom panel of Fig. 8, where four solutions starting from the four different basins are plotted. The solutions are denoted by the letter corresponding to the stable steady state to which they converge.

### Two coupled populations with general coupling

Now we return to the SIS dynamics (4.1) for a general network without forcing symmetry. Specifically, we consider perturbations away from symmetric coupling to gain insight into the dynamics and bifurcations. This allows to exploit the fact that the conditions for bistability remain approximately true as the invariant subspace breaks. Indeed, the hyperbolic part of the branches are preserved while the pitchfork bifurcations may split into bifurcations that appear more generically (saddle-node/fold bifurcations).

As before, we can compute the steady states of the system. For (4.1), these satisfy

4.14a $$\begin{aligned} \tau (d_1y_1 + d_2 y_2)&= \frac{\gamma y_1}{1-y_1} - \beta h_1 y_1^2, \end{aligned}$$

4.14b $$\begin{aligned} \tau (d_3y_1 + d_4 y_2)&= \frac{\gamma y_2}{1-y_2} - \beta h_2 y_2^2. \end{aligned}$$

Dividing the first equation by the second one, we can eliminate $\tau$ and obtain a relation between $y_1$ and $y_2$ as

$$ (d_1y_1 + d_2 y_2) \left( \frac{\gamma y_2}{1-y_2} - \beta h_2 y_2^2 \right) = (d_3y_1 + d_4 y_2) \left( \frac{\gamma y_1}{1-y_1} - \beta h_1 y_1^2 \right). $$

This can be rearranged to

4.15a $$\begin{aligned}&\gamma \left (( d_1-d_4) y_1y_2 +d_2 y_2^2- d_3 y_1^2 + (d_3-d_1)y_1^2y_2+ (d_4-d_2) y_1y_2^2 \right) \end{aligned}$$

4.15b $$\begin{aligned}&\qquad = \beta \left( h_2d_2y_2^3 -h_1d_3y_1^3 + h_2d_1y_1y_2^2 - h_1d_4y_1^2y_2 \right) (1-y_1)(1-y_2).&\end{aligned}$$

These equations can be solved numerically to determine branches of equilibria. Specifically, solve the quartic equation in $y_1$ numerically for a given value of $y_2$. Then (4.14) can be used to compute $\tau$. Hence the equilibrium curve in the $(\tau, y_1)$-plane can be obtained semi-analytically parametrised by $y_2$ as follows. The value of $y_2$ is varied in an appropriate interval and the values of $y_1$ are obtained by solving numerically the quartic Eq. (4.15a). The different solutions of this equation will lead to different branches of the bifurcation curve.

**Fig. 9:**
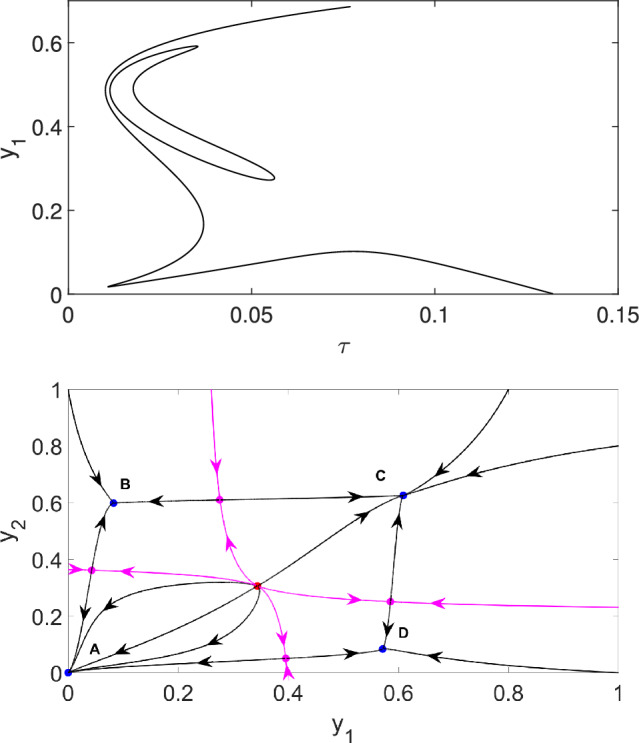
Bifurcations and phase plane for two nonsymmetric populations (4.1) with parameters $d_1=4$, $d_2=3$, $d_3=4$, $d_4=5$, $h_1=1$, $h_2=1.01$, $\gamma =1$, and $\beta =3.85$. Top panel: Branches of equilibria curve in the $(\tau, y_1)$ plane. The bifurcation curve consists of two parts, an isola and an unbounded part. Bottom panel: Phase plane for fixed $\tau =0.03$ shows the stable equilibria A, B, C, and D (blue dots), an unstable symmetric equilibrium (red dot), and four additional (unstable) saddle equilibria (magenta dots). The stable manifolds of the saddle points (magenta curves) separate the basins of attractions of the stable equilibria

To illustrate the dynamics, we used parameter values close to those in Fig. 7; hence this case can be considered as a perturbation of the symmetric case. The bifurcation curve belonging to non-symmetric parameter values is shown in the top panel of Fig. 9. The symmetry $d_1=d_4$ and $h_1=h_2$ of the parameter values is broken by choosing $d_1=4$, $d_4=5$ and $h_1=1$, $h_2=1.01$ that can be considered as a small perturbation of the symmetric case. We can see that the pitchfork curves splits into more generic bifurcations. The number of fold points along the branches remains the same as in the symmetric case but an isola appears in the bifurcation diagram. One can see from the bifurcation diagram that there is a domain of $\tau$ values for which there are 8 non-trivial steady states, three of them are stable, four are saddle points and one is unstable with two-dimensional unstable subspace. The phase portrait corresponding to $\tau =0.03$ is shown in the bottom panel of Fig. 9. This phase portrait is qualitatively equivalent to that shown in the top panel of Fig. 8.

One can observe that there are no foci and centers in the phase plane. The reason for this is that systems (4.1) and (4.2) are monotone dynamical systems. In general, a system $\dot{x} = f(x)$ is monotone, if the sign conditions $\partial _i f_j >0$ hold when $i\ne j$, see Section 3.3 in (Kiss et al. 2017) for more details. Differentiating the first equation of (4.1) or (4.2) with respect to $y_2$ and the second one with respect to $y_1$ yields that this assumption is fulfilled. Monotone dynamical systems cannot have periodic orbits, hence this is true for our systems in general and not only for those parameter values used in Fig. 9. Moreover, trajectories tend to stable nodes for any choice of the parameters.

## Discussion

Reduced mean-field equations give valuable insights into disease spreading on networks that are challenging to obtain from high-dimensional state evolution. Here we focused on a class of models called individual-based mean-field models which arise from considering a bottom-up approach, starting from the evolution equations for the probability of nodes being infected at time *t*. Breaking the dependency on higher-order moments early, results in an *N*-dimensional systems of differential equations which can be simplified to *M* equations as long as nodes in any of the *M* populations are topologically equivalent: The populations themselves can differ but equivalence is necessary within each individual population. Note that the insights we obtain go beyond fully homogeneous populations: Standard hyperbolicity considerations imply that one would expect similar dynamics if the homogeneity is broken (cf. Sect. 4).

The low-dimensional equations we consider are not unique to the reduction approach we used; they also encompass other reduced models proposed in the literature, particularly those involving the application of downward closure. This highlights that one expects to see global dynamical behaviour that is qualitative similar across model equations. The explicit expressions of how the model parameters relate to the network properties gives explicit insights into how network structure—and in particular the presence of higher-order interactions—affect the dynamics. Interaction order has a direct influence on the possibility of multistability for one population: We showed that models with up to three-body interactions can only give rise to either a transcritical transition or bistability and this is in line with findings in Kuehn and Bick (2021). Models with four-body interactions however, show richer behaviour with the previous two possible outcomes being complemented by a multistability regime where two strictly non-zero endemic steady states can co-exist. Furthermore, we investigated a coupled two-population model with up to three-body interactions only. Here, we showed that symmetry breaking, a well studied phenomena in population dynamics, yields a possible route to multistability between endemic steady states. It is clear that the order of interaction affects the degree of the polynomial, with higher-order interactions leading to polynomials of higher degree. This in turn leads to richer behaviours, in our case up to four stable states, and this could present opportunities for further investigation.

Our insights serve as a valuable guide as to what qualitative behaviour one may expect and where (in parameter space) it may arise. Of course, these models only serve as an approximation of microscopic models such as individual-based stochastic models whose number of possible states scale exponentially in the number of individuals. Indeed, given that individual-based mean-field models apply the closure early; at the level of pairs, it means that it misses out some of the important local correlations. Standard results that relate exact and approximate models are typically over finite time (rather than asymptotics); see, for example, Wormald (1995, 1999); Sclosa et al. (2024). One typically expects that good agreement is possible away from bifurcation points. In fact, direct simulations show that the agreement between exact and approximate model can be surprisingly good. While our mean-field models are thus instructive to understand how network structure shapes the contagion dynamics, a direct comparison with stochastic simulations are beyond the scope of this article.

Complex contagion through higher-order interactions shape the dynamics and make it richer compared to classical transmission models. In this paper, we provide a first step towards a mathematical classification of possible dynamics as well as an understanding of the more subtle interactions between structure and dynamics. However, there is further scope to extend this work to addressing questions around overlap between higher-order structures (Malizia et al. 2023, 2023; Burgio et al. 2024) and adaptivity (Burgio, et al. 2023) as well as extension to variations of SIS model by including multiple disease classes leading to models such as SEIS, SIRS or even contact tracing. Furthermore, a fruitful direction is to derive rigorous bifurcation results for 4-body and higher interactions. Note that such extensions should be driven by a well-defined underlying research question, rather than the mere application of existing tools to marginally adjusted models. Finally, we believe that our approach and results underscore the utility of bifurcation theory as a powerful tool for elucidating potential system dynamics and enhancing the understanding of how and why certain outcomes are either promoted or constrained.
